# Psycho-Education on Knowledge of Oral Hygiene and Psychological Distress to the Parents with Leukemia Children

**DOI:** 10.31557/APJCP.2021.22.2.485

**Published:** 2021-02

**Authors:** Ilya Krisnana, Iqlima Dwi Kurnia, Pujiati Pujiati, I Dewa Gede Ugrasena, Yuni Sufyanti Arief

**Affiliations:** 1 *Faculty of Nursing Universitas Airlangga, Surabaya Indonesia. *; 2 *Soetomo General Hospital, Surabaya, Indonesia. *; 3 *Faculty of Medicine Universitas Airlangga,Surabaya, Indonesia. *

**Keywords:** Psycho-education, oral hygiene, psychological distress, leukemia

## Abstract

**Objective:**

To analyze the effect of psycho-educational intervention on knowledge of oral hygiene and psychological distress to the parents of children suffering from leukemia.

**Methods:**

Design of this study was a quasi-experimental pre-posttest control group design. The sample were 70 mothers who had children with leukemia (intervention group = 35 mothers; control group = 35 mothers). The independent variable was psycho-educational, while the dependent variables were oral hygiene knowledge and psychological distress. The instruments used were the knowledge questionnaire and the Depression-Anxiety-Stress Scale (DASS-21). Data were analyzed using the Wilcoxon signed rank test and the Mann Whitney U-test with the significance α =0.05.

**Results:**

The knowledge most widely known by parents was about how to perform of oral care (37.3%). All parameters of knowledge about oral hygiene have increased after being given a psycho-educational intervention. Psycho-educational interventions had an effect on reducing psychological distress; depression (p=0.000), anxiety (p=0.001) and stress (p=0.000).

**Conclusion:**

Most parents whose children suffer from cancer experience psychological distress in the form of depression, anxiety and stress with a range of symptoms ranging from mild to moderate. Psycho-educational interventions can increase knowledge about oral hygiene and decrease psychological distress in parents.

## Introduction

Cancer in children can cause stress (Eldinet al., 2019). Stress occur not only in children, but also in parents. Psychological effects that often arise are anxiety and depression (Al-Maliki et al., 2016), and they can also experience mild to severe stress (Ilya et al., 2019). Stress can be caused due to the malignancy of a child’s illness, the ignorance of how to care for children, and side effects arising from chemotherapy treatment (Rahmani et al.,2018; Rodriguez et al., 2012). Treatment of children, especially those undergoing chemotherapy is largely related to the effects of chemotherapy (Muskat et al., 2017). Oral mucositis is a major side effect in patients receiving chemotherapy (Gandhiet al., 2018; Majoranaet al., 2016). Education about oral hygiene is important for the management of oral mucositis in children with leukemia (Carvalho et al., 2018; Erin, 2015). Psycho-education is a therapy that has been widely used to reduce stress in parents of children with leukemia (Mahmoud and Elaziz, 2015) and several chronic diseases in children (Patraet al., 2015). However, psycho-educational interventions specifically regarding children’s oral care when children get chemotherapy are still limited. 

Around 110 to 130 children cancer cases per million every year. 80% of cases of childhood cancer occur in developing countries. Indonesia as one of the developing countries has incidences of childhood cancer of 11,000 per year (Indonesian Childhood Cancer Foundation, 2017). The most type of cancer in children is Acute Lymphoblastic Leukemia (ALL) that reaches 35.5% (Gandhi et al., 2018). The results of the study showed that ALL ranked first of cancer in children (Krisnana and Purweni, 2018; Krisnana, 2012). Most of the problems that commonly occur in parents related to children with ALL are anxiety with the incidences of 4 out of 5 mothers (Steliarova-Foucher et al., 2017). Based on data from Dr. Soetomo Regional General Hospital in 2017, there were 143 ALL cases and in 2018, there were 114 ALL cases. Dr. Soetomo General Hospital is a type/grade A accredited hospital that functions as a referral hospital in eastern Indonesia. There are several rooms for hematology and oncology treatment of children. The incidence of oral mucositis in Dr. Soetomo Regional General Hospital on children with ALL who get Methotrexate chemotherapy is 62.5% with range of stages from 1 to 4 (Chafid, 2018). 

Parents assume that cancer in children does not only affect the child, but also the whole family (Germannet al., 2018). Stress is felt by parents of children, both fathers and mothers (Deh et al., 2012). Psychoeducation is an alternative choice of intervention that can facilitate psychological conditions in parents (Adkins, 2018). Through psycho-educational interventions, parents can get precise information about how to care for children with cancer related to the side effects of chemotherapy and can explore and give parents the freedom to convey the things that are felt during the treatment process. Parental knowledge related to children’s illness, stress reduction and parents can function properly as a child caregiver (Mahmoud, 2015). In addition, the emotional stability of parents can affect the child’s care for the better result and parents are able to carry out functions and roles in the family (Rahmani et al., 2018). Interventions that involve families can have an impact on increasing children’s adherence to the treatment (Etemadifaret al., 2018). 

## Materials and Methods

*Design*

This research was quantitative research with a quasi-experimental approach. In this quasi-experimental research, the researchers sought to express influences by involving the control group and the experimental group. 

*Sample*

The population in this study was all mothers of children with leukemia in Dr. Soetomo Regional General Hospital that reach 114 people. The inclusion criteria in this study were 1) mothers of children with ALL who received chemotherapy, 2) mothers of children with ALL aged 8-16 years old, 3) mothers who took care of children with ALL directly. Sample size was determined using a formula for two-group research (Dahlan, 2015). The respondents were 32 respondents plus 10% risk of dropping out 3 respondents so that the total respondents per group was 35 people obtained by using a large sample formula. The sample size for the two groups was 70 respondents. The independent variable was psycho-educational interventions, while the dependent variables were oral hygiene knowledge and psychological distress (depression, anxiety, stress). 

*Data collection*

*Administrative and ethical clearance procedures*

The first step in this research was the administration stage in which the researchers asked a research recommendation letter from the Faculty of Nursing. Furthermore, the recommendation letter was addressed to the Director of Regional General Hospital as the requirement of administrative process of the permit for the research site. Next, the Director of Regional General Hospital gave the research permit to the research and development agency to study the ethical conduct of the study. The researchers followed the steps of the ethics due diligence procedure in the form of guiding research appointed to guide the research process. The researchers conducted active monitoring and evaluation because there were interventions aimed at the patient’s family. This research has passed the ethical test by the health research ethics commission of Soetomo Regional General Hospital Surabaya with number of certificate 1610 / KEPK / X / 2019. After the ethics approval letter was issued, the next step was to send a permit to the Head of the Pediatric Ward and a copy to the nurse unit manager of the 1st pediatric ward and 2nd pediatric ward in Soetomo General Hospital where the research was conducted. 

*Selection of respondents*

Prospective respondents were selected according to predetermined inclusion criteria. Based on the formula, the estimated amount needed was 70 respondents divided into two groups namely the treatment group and the control group. The study was conducted in two pediatric hematology wards with the name 1st pediatric ward a treatment group and 2nd pediatric ward as a control group. The researchers gave an explanation of the research (goals, benefits, risks, voluntary participations, etc.) to the prospective respondents in two wards. After the respondents understood and willed to participate in the study, the prospective respondents signed an informed consent. All respondents had completed their informed consent and they were accompanied by the witness signature of the research which is the nurse in the 1st and 2^nd^ pediatric ward. 

*Pre-intervention*

Pre-test was done by giving a questionnaire to the respondents. The questionnaire consisted of knowledge and DASS-21 to measure anxiety levels. Pre-test was done individually and independently by the respondents. The pre-test was done the day before the implementation of psycho-educational interventions. When filling out the pre-test in the form of questionnaire, the researchers entered into a time contract with the respondents for the implementation of the psycho-educational interventions. The schedule for the implementation of psycho-educational interventions was carried out in accordance with the agreement between the researchers and the respondents. 

*Instruments*

The knowledge questionnaire was used to measure the level of knowledge of respondents. The knowledge questionnaire consisted of 12 questions which were divided into 4 subscales, namely 1) definition of oral hygiene, 2) objectives of oral care, 3) benefits of oral hygiene, 4) how to perform oral care in children with cancer. Respondents’ answers were true or false. If the respondent’s answer was true then it was given a score of 1, while the wrong answer was given a score of 0. Maximum score was 12, while the minimum score was 0. The score results were accumulated and then categorized into 3 namely good knowledge (76-100% correct answers), sufficient (56-75% correct answers) and less (<55% correct answers). 

Psychological distress was measured using the DASS-21 questionnaire (Lovibond and Lovibond, 1995). This questionnaire consisted of 21 statements with answers in the form of a Likert scale; never = 0; sometimes = 1; often = 2, almost always = 3. DASS-21 is a set of three self-report scales designed to measure the emotional states of depression, anxiety and stress. Scores on the DASS-21 were multiplied by 2 to calculate the final score. The DASS-21 questionnaire has been translated into Indonesian by Damanik (2011) and has been tested for validity and reliability by Krisnana (2012) and Krisnana et al., (2019) with Cronbach Alpha = 0.97. This figure showed that the questionnaire was declared reliable to measure anxiety because it was more than 0.70 (Heale and Twycross, 2015). 

*Psycho-educational interventions*

Psycho-educational interventions carried out in four stages including 1) education about ALL, 2) management of chemotherapy and prevention of side effects, 3) education about oral hygiene management to prevent the effects of chemotherapy, and 4) stress management through deep breathing relaxation techniques. The four stages in psycho-educational interventions were carried out in at least two meetings. Each meeting consisted of two stages of psychoeducation. The first meeting was about stage 1 and stage 2, while the second meeting discussed about stage 3 and 4. Stages 1 – 3 were done by implementing the lecture method to provide education, while the fourth stage was done by implementing the demonstration method to directly practice deep breathing relaxation techniques. The duration of each stage was 30 minutes. The media used in psycho-educational interventions were in the form of leaflets. Meanwhile, the control group got routine education activities organized by the official of the room.

*Post- intervention*

Post test was conducted on the 6th day. Respondents were given the same questionnaire during the pre-test namely the knowledge and DASS-21. Post-tests were given to both the control and treatment group.

*Data analysis*

Data analysis used by the researchers in this study was descriptive and inferential analysis. Measures of descriptive analysis included the mean, standard deviation, and minimum and maximum values. Meanwhile for the inferential analysis, the researchers used bivariate data analysis. The statistical tests used were Wilcoxon signed rank test and Mann-Whitney U test. Wilcoxon signed rank test was used to analyze differences in data before and after the implementation of psycho-educational interventions in the two groups. While, the Mann-Whitney U test was used to determine the effect of psychoeducation on knowledge of oral hygiene and psychological distress of parents whose children with ALL in Dr. Soetomo Regional General Hospital. The level of significance α=.05. 

## Results

*General characteristics of the participants*

The number of respondents were 70 mothers divided into two groups namely the treatment group (n=35) and the control group (n=35). The two groups had almost the same characteristics. The majority of respondents aged 36-50 years old were divided into two groups in which their majority of educational backgrounds were senior high school. Most of the mothers of both the control group and the treatment group were housewives. The duration of 1-2 months of the children with ALL has the greatest percentage than others (Table 1).

*Level of knowledge*

There are 4 subscales used to measure the level of respondents’ knowledge about ALL and the prevention of side effects of chemotherapy through oral hygiene, namely 1) definition of oral hygiene 2) objectives of oral care, 3) benefits of oral hygiene, and 4) how to perform oral care in children with cancer. Generally, there was an increase in the level of respondent’s ability regarding oral hygiene seen from the number of correct answers in the treatment and control group. Conversely, the parameters concerning the definition of oral hygiene had the smallest percentage after being given psycho-educational interventions in the treatment group. Meanwhile, in the control group, the parameter regarding the objectives of oral care had the lowest percentage at the post test (Table 2).

Knowledge score results obtained were then categorized into three namely 1) good, 2) moderate, and 3) low. The level of knowledge of both control and treatment group was compared to know the comparison before and after being given the psycho-educational interventions. None of the respondents had low knowledge before and after the implementation of interventions in both groups. The level of good knowledge dominated respondents in the treatment group before the implementation of intervention (65.7%). Whereas, the control group was dominated by respondents with the moderate level of knowledge (80%). After the implementation of psycho-educational interventions, both groups experienced an increase in the level of knowledge. All respondents (100%) in the treatment group had a good level of knowledge, whereas in the control group there were still respondents who had a moderate level of knowledge (2.9%). The results of statistical tests indicated that there was an influence of psycho-educational interventions on the level of knowledge of respondents with a value of p = 0<0.001 (Table 3).

*Psychological Distress*

*Depression*

The mean level of depression of respondents before being given psycho-educational interventions was in the range of mild depression which was > 9 (10.80) on treatment group. Meanwhile the average level of depression in the treatment group was at moderate depression level (15.03). After being given a psycho-educational intervention, the mean level of depression in the treatment group dropped to normal level (10.80 to 4.17). Meanwhile in the control group, there was a slight decrease in the mean score and remained at the range of moderate depression (15.03 to 14.29). The results of statistical tests indicated that there was a significant influence of psycho-educational interventions on reducing the level of depression of mothers whose children diagnosed with leukemia (p<0.001).

*Anxiety*

The average level of anxiety of respondents in the treatment group before being given a psycho-educational interventions was at a moderate level of anxiety (11.43). Whereas, in the control group, the mean was 8.51 which was categorized into mild anxiety levels. There was a sharp decrease in anxiety level in the treatment group, which was from the moderate level of anxiety to the normal level. Whereas, in the control group there was a decrease in the mean score that was less significant so that respondents remained at the same anxiety level as the previous one, which was at the level of mild anxiety. The result of statistical tests indicated that there was a significant influence in the provision of psycho-educational interventions on reducing anxiety levels of mothers whose children diagnosed with leukemia.

*Stress*

Similar to depression and anxiety, respondents’ stress levels also decreased significantly after being given a psycho-educational intervention. Stress levels of respondents were at normal level for both respondents in the treatment group and in the control group. However, if it was seen from the decrease in the mean score, the treatment group experienced a greater score reduction than the control group. Based on the results of Mann-Whitney U test, it was obtained a significant p value, which meant that there was an influence of psycho-educational interventions on decreasing anxiety levels of mothers whose children diagnosed with leukemia (p=<0.001) (Table 4).

**Table 1:** Demographic Characteristics (n=70)

Characteristics	Treatment group (n=35)		Control group (n=35)	
	n	%	n	%
Age (years old)				
26-35	17	48.6	13	37.1
36-50	16	45.7	22	62.9
>50	2	5.7	0	0
Level of education				
Elementary	3	8.6	2	5.7
Junior high school	8	22.9	10	28.6
Senior high school	18	51.4	20	57.1
Diploma	3	8.6	3	8.6
Bachelor	3	8.6	0	0
Occupation				
Housewives	26	74.3	33	94.2
Entrepreneurs	3	8.6	1	2.9
Government employer	2	5.7	1	2.9
Farmer	4	11.4	0	0
The duration, children had been diagnosed (months)				
< 1	7	20	2	5.7
1-2	11	31.4	15	42.9
3-4	10	28.6	10	28.6
5-6	5	14.3	6	17.1
> 6	2	5.7	2	5.7

**Table 2:** Level of Knowledge before and after the Implementation of Psycho-Educational Interventions (n=70)

Parameters	Treatment group		Control group	
	Pre-tes	Post-test	Pre-test	Post-test
	Correct (%)	Correct (%)	Correct (%)	Correct (%)
Definition of oral hygiene	28.5	65	44	75
Objectives of oral care	17.1	70	19	30
Benefits of oral hygiene	17.1	70	17	72
How to perform oral care	37.3	90	20	65

**Table 3:** The Effect of Psycho-Educational Interventions on the Level of Knowledge

		Treatment group				Control Group			
	Category	Pre-test		Post-test		pre-test		post-test	
		n	%	n	%	n	%	n	%
Knowledge	Good	23	65.7	35	100	7	20	34	97.1
	Moderate	12	34.3	0	0	28	80	1	2.9
	Low	0	0	0	0	0	0	0	0
Wilcoxon Signed Ranked Test			p=<0.001*			p=<0.001*			
Mann-Whitney U test			p=<0.001*						

*significance on the level α=0.05

**Table 4:** The Psychological Distress before and after the Implementation of Psycho-Educational Interventions

	Treatment group					Control Group					
	Mean		Standart Deviation		p*	Mean		Standart Deviation		p*	p**
	pre-test	Post-test	Pre-test	Post-test		Pre-test	Post-test	Pre-test	Post-test		
Depression	10.8	4.17	7.344	3.044	<0.001	15.03	14.29	6.405	5.555	0.186	<0.001
Anxiety	11.43	5.6	6.37	2.24	<0.001	8.51	8.23	6.007	6.522	0.734	0.001
Stress	11.03	4.51	6.162	2.188	<0.001	11.2	10.86	4.206	4.4	0.797	<0.001

*, significance on Wilcoxon signed rank test; **, significance on Mann-Whitney U test

## Discussion

Most parents who have children with ALL have moderate knowledge in control group and good knowledge in treatment group. The most knowledge parameter which has not yet been known by mother was about the objectives and benefits of oral care. Oral care is one of the management of child care to prevent the side effects of chemotherapy (Northeast Cancer Centre, 2014). Education about oral hygiene is important for the management of oral mucositis in children with leukemia (Boccoliniet al., 2017). Lack of knowledge can increase anxiety and fear in parents with children diagnosed with cancer (Nairet al., 2017). Half of parents whose children having oral mucositis have an impact on psychological well-being (Kamsvet al., 2014)

Psychoeducation is an intervention that can improve knowledge and psychological status of parents’ anxiety related to the conditions of children with cancer (Mahmoud and Elaziz, 2015; Othman et al., 2010). After being given the psycho-educational interventions, respondents’ knowledge about children’s oral care increased. Parental knowledge can be significantly increased due to face-to-face education equipped with leaflet media (Othman et al., 2010). Parents can re-read information that has been given through face to face by reading leaflets that have been given whenever they want to read. Psycho-educational interventions can increase the level of parental knowledge about oral hygiene in children. After being given a psycho-educational intervention, all parents who became respondents had a good level of knowledge. The information provided can be understood very well because the level of education of the respondents is mostly at the level of senior high school. The level of education is closely related to the level of knowledge about the disease and its management (Diaz-Quijano et al., 2018).

Psycho-educational interventions are very useful especially for parents with children who are diagnosed with cancer. Parents with children newly diagnosed with cancer have a high level of anxiety (Patiño-fernández et al., 2008) and high stress levels, so nurses need to facilitate them in managing stress through nursing care (Fernanda, 2013). Psychoeducation also plays an important role in problem solving skills so that it can improve the emotional status of parents including reducing levels of stress, anxiety and depression (Psychological distress) (Tang et al., 2019). 
